# A proposed difficulty grading system for laparoscopic bile duct exploration: benefits to clinical practice, training and research

**DOI:** 10.1007/s00464-023-10169-9

**Published:** 2023-06-22

**Authors:** Ahmad H. M. Nassar, Mahmoud Sallam, Khurram S. Khan, Rhona Kilpatrick, Samer Zino, Tarek Z. Katbeh

**Affiliations:** 1 Laparoscopic Biliary Unit, University Hospital Monklands, Airdrie, Scotland, UK; 2 University of Glasgow, Glasgow, Scotland, UK; 3 University Hospital Hairmyres, Lanarkshire, UK; 4 Department of Surgery, Ninewells Hospital and Medical School, Dundee, Scotland, UK; 5 Department of Surgery, The Queen Elizabeth University Hospital, Glasgow, Scotland, UK; 6 Golden Jubilee National Hospital, Glasgow, Scotland, UK

**Keywords:** Laparoscopic bile duct exploration, Difficulty grading, Tranascystic, Choledochotomy, Impacted bile duct stones, Choledochoscopy, Wiper Blade Manoeuvre WBM

## Abstract

**Background:**

A gap remains between the mounting evidence for single session management of bile duct stones and the adoption of this approach. Laparoscopic bile duct exploration (LBDE) is limited by the scarcity of training opportunities and adequate equipment and by the perception that the technique requires a high skill-set. The aim of this study was to create a new classification of difficulty based on operative characteristics and to stratify postoperative outcomes of easy vs. difficult LBDE irrespective of the surgeon’s experience.

**Methods:**

A cohort of 1335 LBDEs was classified according to the location, number and size of ductal stones, the retrieval technique, utilisation of choledochoscopy and specific biliary pathologies encountered. A combination of features indicated easy (Grades I and II A & B) or difficult (Grades III A and B, IV and V) transcystic or transcholedochal explorations.

**Results:**

78.3% of patients with acute cholecystitis or pancreatitis, 37% with jaundice and 46% with cholangitis had easy explorations. Difficult explorations were more likely to present as emergencies, with obstructive jaundice, previous sphincterotomy and dilated bile ducts on ultrasound scans. 77.7% of easy explorations were transcystic and 62.3% of difficult explorations transductal. Choledochoscopy was utilised in 23.4% of easy vs. 98% of difficult explorations. The use of biliary drains, open conversions, median operative time, biliary-related complications, hospital stay, readmissions, and retained stones increased with the difficulty grade. Grades I and II patients had 2 or more hospital episodes in 26.5% vs. 41.2% for grades III to V. There were 2 deaths in difficulty Grade V and one in Grade IIB.

**Conclusion:**

Difficulty grading of LBDE is useful in predicting outcomes and facilitating comparison between studies. It ensures fair structuring and assessment of training and progress of the learning curve. LBDEs were easy in 72% with 77% completed transcystically. This may encourage more units to adopt this approach.

**Graphical abstract:**

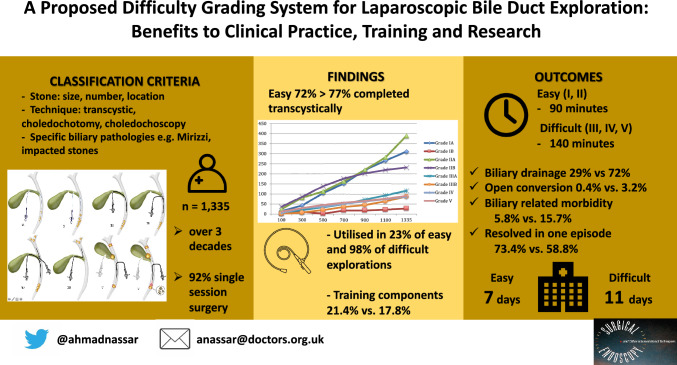

**Supplementary Information:**

The online version contains supplementary material available at 10.1007/s00464-023-10169-9.

The last few years have seen an increasing number of studies reporting single session laparoscopic management of bile duct stones. The guidelines of the National Institute for Health and Care Excellence and those of the Society of American Gastrointestinal and Endoscopic Surgeons (SAGES) suggest that laparoscopic bile duct exploration (LBDE) might be the optimal treatment of ductal stones where experienced surgeons and specialised equipment are available [1, 2]. The Association of Upper Gastrointestinal Surgery of Great Britain and Ireland (AUGIS) emphasises that patients diagnosed intra-operatively with common bile duct stones (CBDS) would ideally have their bile duct cleared simultaneously by LBDE [3].

In spite of the mounting evidence for single session management most surgeons remain reluctant to adopt this approach. The availability of LBDE is limited by the scarcity of training opportunities, by the lack of adequate equipment (fluoroscopy, retrieval baskets, choledochoscopy, additional laparoscopic stacks, dedicated radiographer availability, etc.) and by the perception by some surgeons that the technique of LBDE is difficult. Baucom et al. surveyed surgeons in the United States and concluded that most favoured the staged approach due to lack of comfort performing LBDE [4].

Many studies have been added to the literature in the last few years detailing the efficacy, techniques and outcomes of LBDE. Some have specifically addressed a range of the technical difficulties encountered. However, all lack a clear and objective definition of difficulty. The absence of this important concept may lead surgeons to overestimate the difficulties and challenges they face during LBDE. An objective classification of difficulty would aid accurate assessment of LBDE training, adjusting the outcome parameters according to the case mix and the comparison between studies to inform the reporting of results from different centres. Objective description of the technical difficulties is determined by the pathology encountered and the technique needed to achieve clearance of the bile ducts. Any potential bias resulting from the views of surgeons with different levels of experience and skill would thus be eliminated. The primary aim of this study was, therefore, to establish a difficulty grading system of LBDE according to a five-grade classification [5]. The secondary aims were to study the preoeprative and operative characteristics of easy versus difficult LBDE in a large series, irrespective of the learning curve and the surgeon’s experience and describe the effects of difficulty on the postoperative outcomes.

## Methods

A review of the technical fields of a database of 1335 LBDE performed over 29 years was carried out. All surgeries were performed by a single surgeon (AHMN) or his trainees under direct on-table supervision between 1992 and 2021. The prospectively maintained datasheets were completed after each procedure and the data transferred onto an electronic database (dBase 3 until 1997 then Microsoft Access). Patient demographics, type of admission, clinical presentation, radiological findings, operative time, conversion, complications, hospital stay, readmissions, number of episodes, interval from presentation to resolution and mortality were extracted and analysed.

This specialised unit is dedicated to the management of emergency and elective biliary surgery with occasional referrals from other centres for management of bile duct stones (BDS). All admissions and referrals with suspected gallstones would undergo ultrasound scanning (USS) and routine blood tests including liver function tests (LFTs). Once the presence of cholecystolithiasis is confirmed or preoperative predictors of choledocholithiasis are identified, namely presentation with deranged liver function, jaundice, pancreatitis with deranged liver function or suspected or confirmed stones on preoperative imaging, patients who are deemed fit for general anaesthesia would be offered LC and intra-operative cholangiography (IOC) ± LBDE. Only patients without evidence of gallstones on USS would have magnetic resonance cholangiopancreatography (MRCP). Those with severe pancreatitis or suspected malignancy would have computed tomography scans (CT). Patients who are unfit for general anaesthesia, had a previous cholecystectomy or are severely septic due to severe pancreatitis/cholangitis would have MRCP followed by ERCP and endoscopic sphincterotomy if BDS are confirmed. ERCP is not used for preoperative stone clearance in fit patients at this unit as a matter of protocol. There has been no ERCP service at this hospital for 20 years and all patients requring ERCP (those unfit for surgery,previously cholecystectomised,and any postoperative ERCPs) are carried out at a sister hospital. The service model, preoperative preparation, patient selection, indications for transcystic and choledochotomy exploration, operative features and portoperative outcomes have been published [6].

**Fig. 1:**
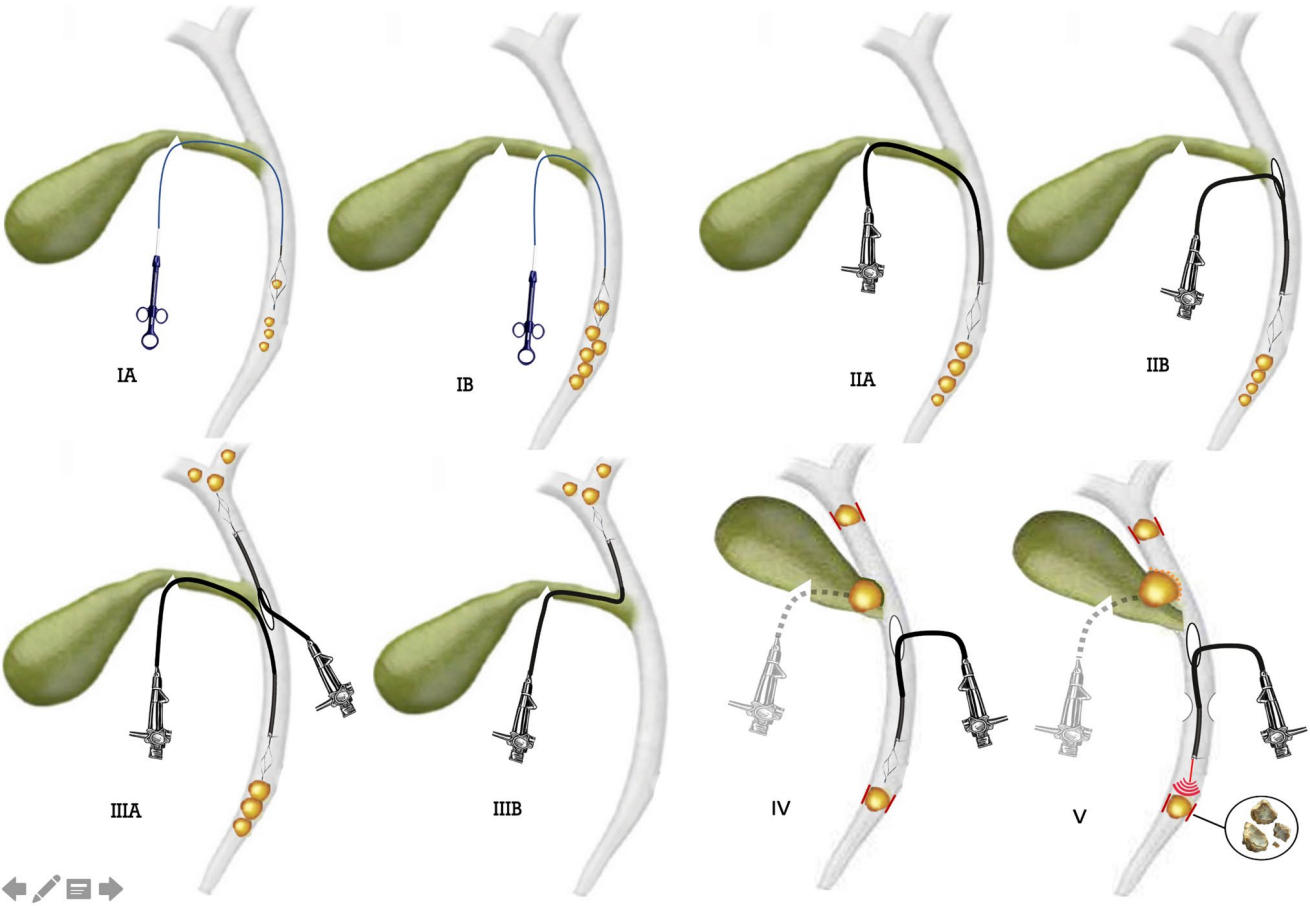
The techniques used for stone retrieval in the different difficulty grades. **IA** Transcystic blind basket trawling for a few distal CBD stones < 8 mm in diameter. **IB** Transcystic basket extraction of 1–10 stones 8–10 mm in diameter requiring further proximal cystic duct incisions. I**IA** Transcystic choledochoscopic stone extraction. **IIB** Transductal choledochoscopic stone extraction. **IIIA** Choledochoscopic exploration for 1–15 stones up to 15 mm in diameter; either transcystic for distal stones or transductal for CBD or intrahepatic stones. **IIIB** Transcystic choledochoscopic Wiper Blade Manoeuvre for intrahepatic stones of any number and any size. **IV** Choledochoscopic TCE for Mirizzi Type I or transductal exploration for impacted stones (any number or size) needing dislodging manoeuvres or antegrade stenting required. **V** Trans-fistula exploration for Mirizzi II or choledochotomy for impacted stones needing fragmentation/ open conversion / bilioenteric anastomosis e.g. Mirizzi III or IV

The operative details recorded on the original data sheet were examined to retrospectively assign a difficulty grade to each procedure. LBDE procedures were graded according to stone-related parameters; the size of stones; number of stones; location of stones in biliary tree (cystic duct CD, common bile duct CBD, intrahepatic and common hepatic duct CHD); the exploration technique (transcystic exploration TCE or choledochotomy); the utilisation of choledochoscopy and specific biliary pathologies encountered. Each exploration was graded according to the presence of two or more of the above difficulty criteria, with the techniques required to extract the stones reflecting the complexity of the procedure (Fig. 1). Difficulty factors related to access e.g. previous upper abdominal surgery, adhesions, inflammatory conditions or cholecystoenteric attachments were excluded as these had no direct effect on the technical difficulty of the exploration.

LBDEs were classified into five grades (Table 1) and, for the purpose of meaningful analysis, divided into two groups; Easy LBDE (grades IA, IB, IIA and IIB) and Difficult LBDE (grades IIIA, IIIB, IV and V).

**Table 1:** Difficulty grading classification for laparoscopic bile duct exploration

Difficulty grade	Stone location	Stone number	Stone size mm	Technical factors
Grade IA	CBD	1–5	< 8	TCE, blind Basket-in–Catheter (BIC) technique
Grade IB	CBD	1–10	8–10	TCE, blind BIC; CD diameter or configuration hinder cannulation, requring further proximal incision/dilatation. Or X-Ray guidance needed to engage stones
Grade IIA	CBD	1–10	8–15	Choledochscopic TCE or Stone recovery from CD stump needs crushing or further CD incision. cystic anomalies (low or medial implantation)
Grade IIB	CBD	1–15	Up to 15	Choledochotomy with or without biliary drainage or TCE with biliary drainage
Grade IIIA	CBD or CHD	> 15	Up to 15	Choledochoscopic TCE for large distal stones or choledochotomy for large CBD or intrahepatic stones
Grade IIIB	CHD	Any	Any	TCE with choledochoscopic WBM stone retrieval
Grade IV	Any	Any	Any	TCE for Mirizzi type I/choledochotomy for impacted stones needing dislodging manoeuvres/need for antegrade stenting
Grade V	Any	Any	Any	TCE; transfistula exploration for Mirizzi II/CBDE for impacted stones needing fragmentation/open conversion/bilioenteric anastomosis e.g. Mirizzi Types III or IV

*CBD* common bile duct, *CHD* common hepatic duct, *TCE* transcystic exploration, *BIC* basket-in-catheter, *CD* cystic duct, *WBM* wiper blade manoeuvre

Informed consents for LC and LBDE if indicated had been obtained from all patients. The database was registered in the audit department. Ethical approvals was not required as the unit’s protocols were consistent with national and international guidelines.

Most patients were followed up within 3 months and annually thereafter (range 3 months to 24 years). Electronic records were reviewed of the last 1000 patients over three months in 2020, due to the impact of COVID pandemic, to complete the follow up fields on the database and to spot evidence of recurrent stones or biliary-related complications.

## Statistical analysis

Data was analysed using IBM SPSS software package version 20.0. (Armonk, NY: IBM Corp). The Kolmogorov–Smirnov was used to verify the normality of distribution of variables. Comparisons between groups for categorical variables were assessed using Chi-square test. Significance of the obtained results was judged at the 5% level. Statistically significant results are considered at *p* value ≤ 0.05.

## Results

**Fig. 2:**
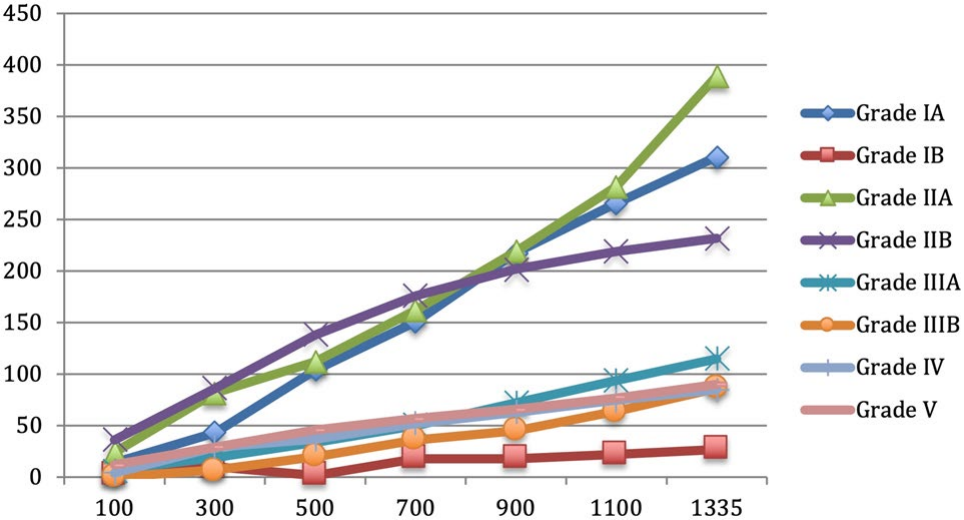
The changing incidence of individual LBDE difficulty grades vs. the total number of cases as the series progressed

1335 LBDE were included in a series of 5780 cholecystectomies (23.1%). 959 (71.8%) were “easy” explorations, classified as Grades IA and B and IIA and B. 376 (28.2%) explorations met the criteria for Grades III, IV, V and were classified as “difficult”. The changing incidence of individual LBDE difficulty grades in relation to the total number of explorations at landmarks of increasing experience as the series progressed is shown in Fig. 2. The utilisation of transductal exploration is shown to decline at the half-way point in favour of a significant increase in transcystic explorations. There was also an increase in successful transcystic intrahepatic choledochoscopy, so called the Wiper Blade Manoeuvre (WBM), Grade IIIB.

Approximately two thirds of the patients in both groups were females. The mean and median age were comparable in the two groups (Table 2). As would be expected, previous ERCP is associated with increased operative difficulty.

**Table 2:** Demographic features and preoperative data in easy vs. difficult LBDE

	Easy (Grade I A, B and II A, B) *N* = 959 (71.8%)	Difficult (Grade III, IV, V) *N* = 376 (28.2%)	*p* value	OR (95% CI)
Sex female	647 (67.5)	243 (64.6)	0.322	1.135 (0.883, 1.459)
Median age (years)	59 (IQR 44–70)	64 (IQR 49–74)	** < 0.001**	–
Emergency admission	688 (71.7)	317 (84.3)	** < 0.001**	0.473 (0.346, 0.645)
Presentation*				
Acute pain	316 (33)	245 (65)	** < 0.001**	0.263 (0.204, 0.338)
Acute cholecystitis	73 (7.6)	21 (4)	0.193	1.393 (0.844, 2.298)
Acute gallstone pancreatitis	115 (12)	31 (8.2)	**0.048**	1.516 (1.000, 2.299)
Jaundice	427 (44.5)	254 (67.5)	** < 0.001**	0.386 (0.300, 0.495)
BMI ≥ 35	60 (6.2)	32 (8.5)	0.144	0.717 (0.459, 1.121)
Previous episodes				
Total	294 (30.6)	111 (29.5)	0.685	1.055 (0.813, 1.370)
At other units	144 (15)	84 (22.3)	**0.001**	0.614 (0.455, 0.829)
Preoperative imaging				
MRCP	145 (15.1)	75 (19.9)	0.032	0.715 (0.525, 0.973)
Previous ERCP	46(4.8)	62 (16.5)	** < 0.001**	0.255 (0.171, 0.382)
Ultrasound				
Multiple gall stones	855 (89)	314 (83.5)	**0.005**	1.623 (1.155, 2.281)
Single stones/none	52 (5.4)	26 (6.9)	0.296	0.772 (0.474, 1.256)
Contracted/thick wall	158 (16.4)	69 (18.3)	0.412	0.878 (0.642, 1.199)
CBD dilated	407 (42.4)	250 (66.5)	** < 0.001**	0.372 (0.290, 0.477)
Preoperative predictors of CBDS **	737 (76.8)	351 (93.3)	** < 0.001**	0.236 (0.153, 0.364)

Bold values indicate significant

*BMI* Body mass index, *MRCP* Magnetic resonance cholangiopancreatography, *ERCP* Endoscopic retrograde cholangiopancreatography, *CBD* Common bile duct, *CBDS* common bile duct stone

*More than one presentation may be entered e.g. acute pain + jaundice

**Presentation with deranged liver function, jaundice, pancreatitis, suspected or confirmed stones on preoperative imaging

Obesity and previous admission episodes were not associated with significant differences between easy and difficult explorations (p = 0.144 and 0.685 respectively). However, a previous admission at other hospitals was significantly associated with difficult explorations as most patients were referred after attempted endoscopic clearance and occasionally with recurrent stones after previous biliary surgery.

### Predictors of difficult exploration

Preoperative predictors or risk factors for CBD stones were recorded in 76.8% of easy vs. 93.3% of difficult explorations (*p* < 0.001). Specific predictors were associated with difficult explorations. Difficult explorations were more likely than easy explorations to have been emergency admissions (84.3% vs 71.7%, *p* < 0.001). Presentation with acute pain with or without jaundice accounted for nearly two thirds (65%) of difficult explorations compared to 427 (44.5%) of easy explorations. Of all patients undergoing ductal explorations only 10.9% presented with acute pancreatitis and 7% with acute cholecystitis. However, most of these (78.7% and 77.7% respectively) had easy explorations.

Those who had undergone previous endoscopic sphincterotomy, with or without biliary stents, accounted for 62 (16.5%) of difficult explorations compared to 46 (4.8%) of easy explorations, (*p* < 0.001). Of the 108 patients in this cohort who had had one or more failed preoperative ERCPs 90 (83%) had been carried out at other units before they were referred for surgical treatment at this unit or at the referring institution. The remaining 18 (17%) were initially admitted under the biliary unit but were unfit for surgery during the index admission. Further analysis comparing the 108 patients who had previous ERCP and 1227 who underwent single session management showed the superiority of the latter approach (Supplemental Table 1). It is associated with significantly easier explorations, a higher incidence of transcystic exploration with less reliance on choledochoscopy or biliary drainage and shorter operative time and total hopsital stay. 73% resolved completely in one episode vs. 27.7% of the ERCP group and the interval between presentation and episode resolution was much shorter; 62.3% resolving within two weeks vs 20.3% for those who had previous ERCP.

Preoperative USS showing a dilated CBD was seen in 250 (66.5%) of the difficult explorations and in 407 (42.4%) of easy explorations, (*p* = 0.0001).

### Operative findings

The finding of a dilated cystic duct at the time of surgery resulted in a higher LBDE difficulty grade (*p* < 0.001).

The majority (77.7%) of CBD stones were cleared by transcystic exploration in grade I and II cases, whereas choledochotomies were required in 62.3% of grades III-V (difficult) explorations vs. 33.6% in the whole series. (Table 3).

**Table 3:** Operative data and technique

	Easy (grade I A, B and II A, B) *N* = 959 (71.8%)	Difficult (grade III, IV, V) *N* = 376 (28.2%)	*p* value	OR (95% CI)
Cystic pedicle				
Cystic duct stone/s	293 (30.5)	114(30.3)	0.934	1.011 (0.780, 1.310)
Wide cystic duct	286 (29.8)	189 (50.2)	** < 0.001**	0.420 (0.329, 0.537)
Exploration technique				
Transcystic exploration	745 (77.7)	142 (37.7)	** < 0.001**	5.737 (4.432, 7.426)
Choledochotomy	214 (22.3)	234 (62.3)		
Median number of CBD stones	2 (IQR 1–3)	3 (IQR 1–7)	** < 0.001**	–
Median size of largest stones (mm)	7 (IQR 5–9)	12 (IQR 8–15)	** < 0.001**	–
Use of Glucagon	482 (50.2)	124 (33)	** < 0.001**	2.054 (1.600, 2.635)
Choledochoscopy	225 (23.4)	369 (98.1)	** < 0.001**	0.006 (0.003, 0.012)
Biliary drainage				
T-tube	90 (9.4)	137 (36.4)	** < 0.001**	0.181 (0.134, 0.244)
Transcystic tube	188 (19.6)	109 (29)	** < 0.001**	0.597 (0.454, 0.786)
No biliary drain	680 (70.9)	105 (27.9)	** < 0.001**	6.290 (4.827, 8.199)
Stent	1(0.1)	17 (4.5)	** < 0.001**	0.022 (0.003, 0.166)
Biliary bypass	0	8 (2.1)^@^	** < 0.001**	–
Abdominal drain	773 (80.6)	357 (95)	** < 0.001**	0.221 (0.136, 0.360)
Open conversion*	4 (0.4)	12 (3.2)	** < 0.001**	0.127 (0.041, 0.396)
Training components	205 (21.4)	67(17.8)	0.147	1.254 (0.923, 1.703)
Median operative time (minutes)	90 (IQR 70–120)	140 (IQR 110–195)	** < 0.001**	–

Bold values indicate significant

*3 of 16 open conversions were due to adhesions unrelted to bile duct exploration

^@^Including biliary enteric anastomosis in two cases of Mirizzi Types 3 and 4 carried out at liver units as second procedures

Choledochoscopy was utilised in 98.1% of difficult explorations and in only 23.4% of easy explorations.

Biliary drainage was utilised in most of the difficult explorations, either with T tubes (36.4%) or transcystic tubes (29%). Most of the easy explorations did not require any form of biliary drainage (70.9%). The rate of using abdominal drains was also significantly proportional to the difficulty grade.

The overall conversion rate was 1.2% (16/1335). 12 (3.2%) of difficult exploration and only 4 (0.4%) of easy explorations, most in the early phase of the series, had to be converted to open surgery. Three of these resulted from abdominal adhesions and were not directly related to bile duct exploration. As would be expected, the operative time was significantly longer the more difficult the exploration (median time 140 min vs 90 min for easy explorations, *p* < 0.001). Further analysis was carried out to study how the operative time changed over the three decades of the study (supplemental Fig. 1). The surgery time significantly decreased with increasing experience and the refinement of the techniques over time. The operative time was considered out of the normal range (longer than six hours) in only 8 patients(5.9%) where completing surgical clearance, including stone fragmentation and laser, was the only option. Each patient had combined difficulty factors including large impacted stones (7), multiple intrahepatic stones (4), Mirizzi Syndrome (3) and multiple previous failed ERCPs with stents (3).

Table 4 shows the relationship between outcome parameters and LBDE difficulty grading. The overall morbidity rate and the Clavien-Dindo classification of all postoperative complications in this series have previosly been reported [6]. Specific biliary-related complications such as bile leakage, those related to biliary drains and retained stones were significantly higher in the difficult group as compared to easy explorations (5.8% vs 15.7%) as was the incidence of readmissions (6.5% vs 15.9%). There was no significant difference in the rate of reoperation between the two groups. Reoperation was required in 13 patients for complications (8 easy and 5 difficult). Another patient had a Mirizzi Type IV abnormality discovered intraoperatively where, apart from the offending stone, a stone was removed from the lower CBD. Biliary reconstruction was carried out at a liver unit the following day.

**Table 4:** Perioperative outcomes and follow up

	Easy (grade I A, B and II A, B) *N* = 959 (71.8%)	Difficult (grade III, IV, V) *N* = 376 (28.2%)	*p* value	OR (95% CI)
Biliary-related complications				
Bile leakage	7 (0.7)	15 (4)	** < 0.001**	0.177 (0.072, 0.438)
Pancreatitis	9 (0.9)	6 (1.6)	0.305	0.584 (0.207, 1.653)
Hyperamylasemia	16 (1.6)	10 (2.6)	0.238	0.621 (0.279, 1.381)
Retained stones	14 (1.4)	14 (3.7)	**0.009**	0.383 (0.181, 0.811)
Biliary drain related*	10 (1)	14 (3.7)	** < 0.001**	0.272 (0.120, 0.619)
Readmissions*	63 (6.5)	60 (15.9)	** < 0.001**	0.370 (0.254, 0.539)
Reoperations**	8 (0.8)	5 (1.3)	0.407	0.624 (0.203, 1.920)
Total hospital stay (days)***	7 (IQR 4–11)	11 (IQR 7–16)	** < 0.001**	–
Total hospital episode(s)				
1 episode	704 (73.4)	221 (58.8)	** < 0.001**	1.936 (1.507, 2.487)
2 episodes	222 (23.1)	127 (33.8)	** < 0.001**	0.591 (0.455, 0.767)
3 episodes	27 (2.8)	20 (5.3)	**0.026**	0.516 (0.286, 0.931)
≥ 4 episodes	6 (0.63)	8 (2.1)	**0.015**	0.290 (0.100, 0.840)
Presentation to resolution interval (weeks)*	2 (IQR 1–3)	3 (IQR 2 -5)	0.070	–

Bold values indicate significant

*Dehydration due to fluid loss and pain after tube removal included under readmissions

**Plus one for Mirizzi type IV reconstruction. After uncomplicated grade V exploration

***For all hospital episodes including at other hospitals and referring units

The total hospital stay was significantly longer after difficult explorations with a mean of 11 days vs. 7 days for easy explorations (*p* < 0.001). As shown in Table 4 the hospital stay was calculated for all hospital episodes, including admissions at other referring hospitals or, occasionally, units in other countries before the patients underwent surgery. It also included hospital stay during occasional readmissions. Significantly fewer Grades I and II (26.6%) had two or more total admissions, including postoperative readmissions, compared to grades III to V (41.2%). However, the median presentation to resolution interval was not significantly different.

The incidence of the perioperative outcomes in the easy vs. difficult exploration groups correlated with their observed incidence in individual LBDE difficulty grades (Table 5). As would be expected biliary-related complications, median operative time, open conversion, readmissions, retained stones, median hopsital stay and number of hospital episodes increased with higher difficulty grades.

**Table 5:** Perioperative outcomes in individual LCBDE difficulty grades

Grade/number/%	IA and B 338 (25.3%)	IIA 384 (28.7%)	IIB 237 (17.7%)	IIIA 115 (8.6%)	IIIB 86 (6.4%)	IV 85 (6.36%)	V 90 (6.7%)
Complications total	31 (9.2)	32 (8.3)	41 (17.2)	33 (28.7)	12 (14)	17 (20)	25 (27.7)
Biliary-related	20 (5.9)	10 (2.6)	20 (8.4)	20 (17.4)	5 (5.8)	11 (12.9)	19 (21.1)
General	11 (3.2)	22 (5.7)	21 (8.8)	13 (11.3)	7 (8.1)	6 (7)	6 (6.6)
Open conversion	2 (0.6)	0	2 (0.8)	1 (0.8)	0	0	11 (1.1)
Operative time/min							
Mean	80.6	99.3	132	142	130	156	219
Median	70	85	150	120	110	140	180
Range	40–265	42–285	50–310	55–350	60–280	60–570	85–630
Readmissions	17 (5)	14 (3.6)	32 (13.5)	22 (19)	8 (9.3)	16 (18.8)	14 (15.5)
Reoperations	1 (0.03)	2 (0.05)	5 (2.1)	1 (0.08)	0	1 (1.1)	3 (3.3)
Retained stones	4 (1.18)	1 (0.26)	9 (3.8)	4 (3.48)	1 (1.16)	0	9 (1.0)
Hospital stay*							
Mean	6.8	8.7	12.3	16.4	11.5	14	14
Median	4	6	14	10	7	11	**12**
Hospital episodes*							
Median	1	1	2	1	1	1	2
1	271 (80)	289 (75.2)	144 (60.7)	63 (54)	59 (68.6)	50 (58.8)	49 (54.4)
2	56 (16.5)	86 (22.4)	80 (33.7)	43 (37.4)	23 (26.7)	30 (35.3)	31 (34.4)

*For all hospital episodes including at other hospitals and referring units

There were three 30-day deaths in this series (0.2%), all ASA3. One was referred following a failed endoscopic clearance and a stent fully within the CBD. The 60 year old female, with significant peripheral vascular disease, had been in another hospital for three weeks before referral and suffered massive mesenteric ischaemia and bowel infarction four days after an uneventful 135 min LBDE (Grade IIB). An 81 year old male was in hospital for three weeks with cholangitis and jaundice under the care of the physicians. Endoscopic clearance was declined due to a large stone in the common hepatic duct. He was found to have a Mirizzi Type II abnormality and underwent an uncompliacted 270 min Grade V exploration but died of pneumonia three weeks later. A 78 year old female who had a Grade V exploration for Mirizzi Type II also died three weeks postoperatively of pneumonia.

## Discussion

Single session management of bile duct stone was the standard treatment in the open surgery era. Laparoscopic surgery brought about a rapid expansion in laparoscopic cholecystectomy while the technical skills, the equipment and the logistics necessary for intraoperative cholangiography and bile duct exploration lagged behind. This delay in adopting the laparoscopic approach to bile duct stones was multifactorial. The lack of training opportunities, limited availability of adequate equipment (fluoroscopy, retrieval baskets, choledochoscopy, and additional laparoscopic stacks), limitations to operating theatre scheduling and dedicated radiographer input and, more importantly, the perception some surgeons have that the technique of LBDE is difficult favoured the two session approach. However, the adoption of the surgical approach seems to be increasing and relatively large numbers of centres are reporting their techniques and results. A recent multicentre survey of 17 centres in 9 countries reported 3950 bile duct explorations, including a major contribution by this unit’s series, and concluded that LBDE is safe and effective when performed by experienced teams [7]. As a number of international societies agree that in the presence of the expertise and equipment, the results of the laparoscopic approach can be superior to the staged management, the authors recommended the development of more training programs.

One of the obstacles to making training more available is the reluctance of surgeons in most centres to adopt LBDE. The perception that the technique is difficult seems to be a major contributary factor [4].

Specific factors can result in technical difficulties during LBDE, independent of the difficulties attributed to the cholecystectomy. As a significant proportion of transcysctic explorations are carried out using blind basket trawling of the bile duct, the current authors found that the cystic duct diameter and configuration can increase the difficulty in cannulating the duct and accessing the CBD [6]. Special measures are needed to overcome difficult cystic duct configurations, such as further dissection and more proximal incisions, dilatation of the cystic duct and using the Basket-in-Catheter (BIC) technique. Intrahepatic stones may be difficult to access or remove. They would normally need the transcystic choledochcoscopic Wiper Blade Manoeuvre or performing a choledochotomy [8]. When the CD anatomy was difficult Fang et al [9] reported a T shaped incision of the CD towards the CBD junction to facilitate cannulation with laser lithotripsy to fragment and remove large stones. Such measures, however, may not be adequate to overcome certain CD/CBD junction configurations. A low or medial implantation may make it necessary to avoid blind basket exploration attempts and to utilise transcystic choledochoscopy in order to avoid retained stones in the intramural CD or allowing stones to migrate proximally. This can lead to failure of the transcystic approach as the WBM is virtually impossible to perform with such configuration. Resorting to a choledochotomy would then be necessary but may occasionally result in incising into the intramural CD and having difficulty accessing the intrahepatic ducts.

Whether the exploration is carried out transcystically or through a choledochotomy most authors agree that large, numerous, intrahepatic and impacted stones result in a more difficult exploration.

The concept of diffculty scoring as a means of producing more meaningful data and enabling objective analysis of outcome parameters of various methods of treating bile duct stones has been addressed by endoscopists and by liver surgeons. Schuts et al. [10] suggetsted a grading score for endoscopic retrograde cholangiopancreatography (ERCP) by degree of difficulty. A scoring system for laparoscopic liver resection in cases of difficult intrahepatic duct stones (IHD) was proposed by Kim et al. [11].They compared potential factors that can lead to a longer operation time in laparoscopic liver surgery for IHD stones between patients with longer than median operative time and those with shorter operation time. A longer operation time was associated with stone location, extent of liver resection, atrophy of liver parenchyma, ductal stricture < 1 cm from the bifurcation, and the need for combined choledochoscopic examination of the intrahepatic duct remnant. The score was obviously specific to liver resection and would not be applicable to primary ductal exploration.

It has been recognised that the higher the stones in the biliary tree the more difficult their retrieval would be [12]. Kao et al. [13] found that the presence of multiple CBD stones diagnosed at operation almost halved the odds risk of failure.

Suwatthanarak et al [14] recognised the fact that most choledochotomy explorations were relatively easy and reported their use of a technique well known from the open surgery era and used frequently in laparoscopic exploration, describing it as the “Chopstick Technique”, the simple manipulation of the CBD with two instruments. Additional surgical instruments were needed in difficult cases resulting from an increased number of stones, previous ERCP and previous abdominal surgery. They recognised impacted stones as a cause of difficulty, which is a major factor in the current authors experience [6]^.^ However, the main factor in their high conversion rate of 28% was previous abdominal surgery which is unrelated to the LBDE technique.

Impacted stones and intrahepatic stones were also reported to cause failed extraction. Electrohydraulic lithotripsy [15] and holmium laser lithotripsy [16] were used to fragment difficult extrahepatic or intrahepatic stones, avoiding adverse outcomes.

Ma et al [17] recently recognised the lack of reports on the safety and efficacy of LBDE in patients with difficult biliary stones. They attempted to define the criteria for difficulty in a large series of 1064 bile duct explorations. Explorations for large (> 15 mm), multiple (> 3), intrahepatic or impacted biliary stones, as well as those with Mirizzi’s syndrome were defined as difficult and accounted for 334 explorations (31.4%). Interestingly, the incidence of criteria of difficulty was relatively similar to those in our classification, where 28.2% are classified as difficulty grades III, IV and V. Ma et al. defined multiple, more than 3, stones as difficult vs. 15 stones in our series and may, therefore, have upgraded some easier explorations to difficult. They also included criteria of difficulty identified at preoperative ERCP; namely periampullary diverticulum, Rouxen-Y gastric bypass, Billroth-II anatomy, duodenal stricture.

In this study, traditional predictors of difficult cholecystectomies i.e. male sex, older age and obesity did not seem to increase complexity when performing ductal exploration. The preoperative factors found to be predictive of difficult ductal explorations include emergency admission, jaundice, previous ERCP and CBD dilatation or stones on ultrasound scanning. Despite the relatively small numbers of patients undergoing preoperative ERCP in this series these accounted for significantly more difficult explorations. The ability to predict a difficult exploration is helpful for operative planning; deciding the level of experience of the operating surgeon, preparation of equipment and the scheduling of cases.

At operation, previous abdominal surgery would clearly make access to the CBD more difficult. Li et al [18] identified prior abdominal operations and previous biliary tract surgery as significant challenges for LBDE because of the surgically altered gastrointestinal anatomy and adhesions. However, the current classification addresses the technique of ductal exploration and, therefore, excluded difficulties of access caused by postoperative adhesions as these are included in our LC difficulty scale [19]. While it may be difficult to identify the CBD in occasional cases, certain anatomic markers, such as the cystic duct stump or duodenal bulb, may guide the dissection. Direct puncture and aspiration of bile can confirm the structure to be the CBD. Once the exploration is commenced, it may be relatively easy to remove single or multiple stones with the use of a choledochoscope and the exploration, therefore, may not be considered difficult.

Difficulty may also be anticipated when encountering abnormalities of the cystic pedicle or when the cystic duct is dilated. However, the presence of stones in the cystic duct did not seem to add to the complexity of the exploration.

Most easy explorations were completed transystically. However, nearly one quarter of easy explorations were choledochotomies and 142 (37.7%) of the difficult explorations were transcystic.

The success of the utilisation of Glucagon, to relax the Sphincter of Oddi during TCE, was associated with significantly more easy explorations, where stone fragmentation occured. Choledochoscopy is usually required for explorations involving multiple, intrahepatic and impacted stones. Therefore it is expected to be part of the more difficult explorations. Similarly, the need for biliary drainage in this unit, whether temporary with transcystic or T-tubes, or permanent vis bilioenteric anastomosis on a few occasions early in the series, increased with difficulty. Although the conversion rate is significantly higher in difficult explorations only two of the 16 conversions occurred in the last 1164 explorations, after the first decade of the series, and were the result of difficult impacted stones and Mirizzi Type II Syndrome.

Retained stones were found in 3.7% of difficult explorations v.s 1.4% after easy explorations (*p* = 0.009). This is not surprising, considering that most retained stones followed explorations for impacted, large, numerous or intrahepatic stones.

The lack of standardisation of bile duct exploration techniques may also contribute to increased difficulty. Suboptimal positioning of operating ports can make a relatively easy operation more difficult. A lower than necessary right subcostal port may render cholangiography and choledochoscopy challenging due to difficulty of cystic duct cannulation. Attempting to insert the cholangiography catheter or choledochoscope through the epigastric port or a port in the left upper quadrant or without an introducer can also complicate the transcystic insertion of a choledochoscope or render effective manipulation difficult. Difficulty increases when employing additional technical steps such as the use of guide wires or dilators of the cystic duct. The complete separation of the gallbladder from the liver and routine dissection of the cystic duct/CBD junction [20] have also been described. The configuration of the cystic duct/CBD junction may still render such steps ineffective in accessing the CBD. The routine use of laser lithotripsy has been advocated by some authors as a means of maximising the rate of transcystic exploration [21]. While such examples may occasionally be useful in some experienced units, their regular use adds to the difficulty and operative time. They have the potential for adding to the difficulty in performing ductal explorations, at least in the early phase of the learning curve of the inexperienced surgeon. Individual preferences which increase the complexity of the exploration risk adding to the obstacles to the wider adoption of laparoscopic bile duct exploration and have, therefore, not been considered in the current classification of difficulty.

## Limitations

**Fig. 3:**
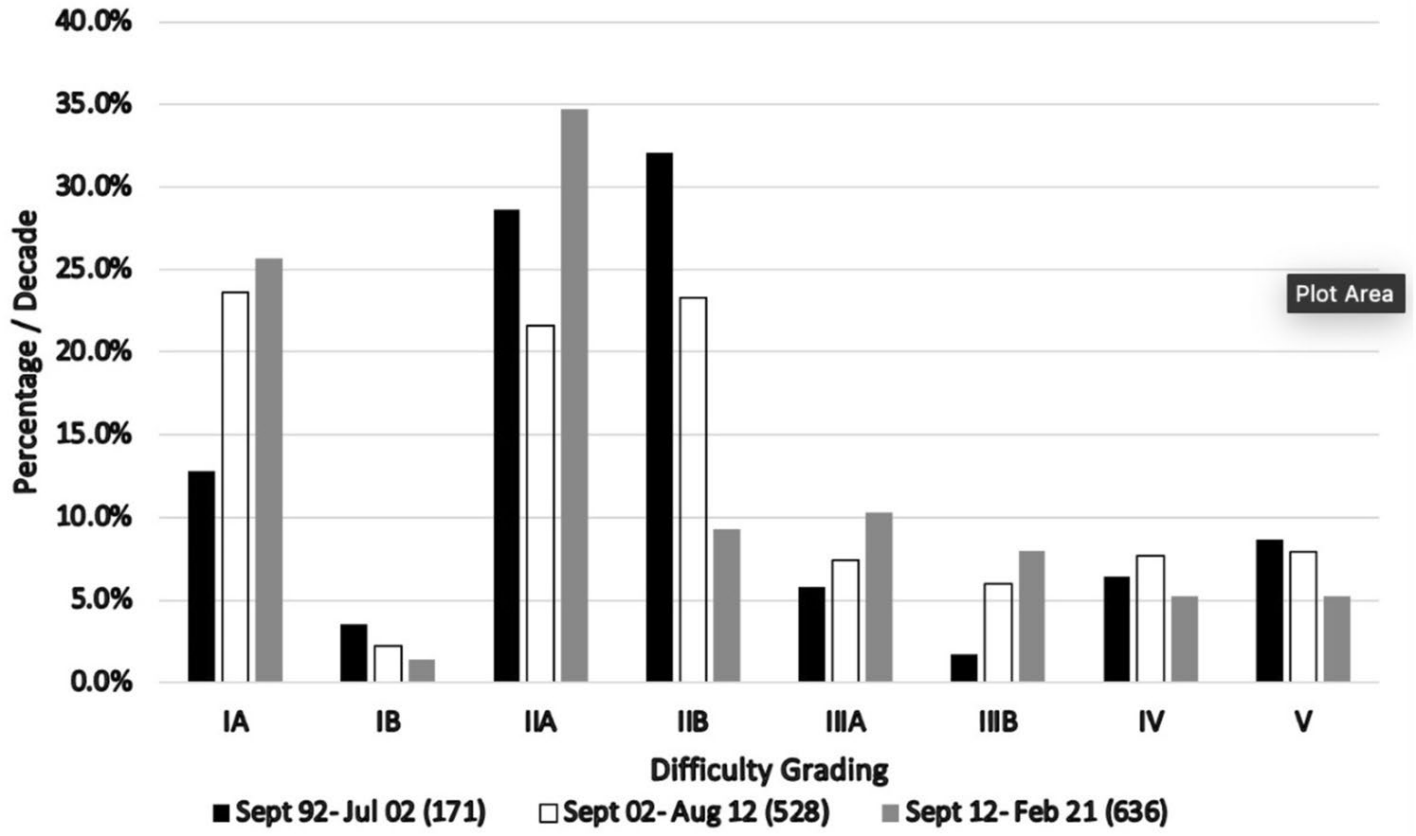
The effect of increasing experience over the three decades of the study on the incidence of individual difficulty grades

This is a single surgeon series stretching over 30 years. Inevitably, some technical aspects were refined during this time. The classification was based on all bile duct explorations with no exclusions. Although some were performed early in the experience, the effects of the learning curve on the classification were mitigated by the large size of the series. The increasing experience over the period of the study resulted in a higher success rate of transcystic clearance in Gardes IA, IIA, IIIA and IIIB and a reduction in resorting to choledochotomy in Grades IIB, IIIA, IV and V as shown in Fig. 3. This understandably reduced the incidence of the most difficult explorations; Grades IV and V, with the passage of time although most grading cirteria remianed unchanged. Increased utilisation of transcystic exploration reducing the difficulty over time can therefore be used to monitor performance and assess the progress of training. Most classifications require a large retrospective dataset to allow wider evaluation of the trends and more logical extrapolation of the criteria. This classification remains applicable to large prospective series from different centres with varying levels of experience and different technical methodology that would allow meaningful validation.

## Conclusion

The proposed difficulty grading classification for laparoscopic bile duct exploration, based on the largest reported series, offers practical benefits. It identifies certain preoperative predictive criteria, helping surgical planning. Operative difficulty features may influence the surgeon’s adoption of special techniques and instruments, facilitate important decision making e.g. whether to utilise biliary drains, and allow accurate and standardised reporting of the operative findings. This helps to identify patients at higher risk of complications, allowing the modification of postoperative management protocols, thus optimising important outcome parameters. As the adoption of single session management of bile duct stones gains more popularity difficulty grading will be an important tool in the objective assessment of the progress of training and in comparing studies. Validation of the classification through prospective series of bile duct explorations from different centres is required.

## Supplementary Information

Below is the link to the electronic supplementary material.

Supplementary file1 (DOCX 21 KB)

Supplementary file2 (JPG 45 KB) Supplemental Fig. 1 The effect of increasing experience on the operative time in easy v.s difficult LBDE over the three decades of the study

## References

[1] Internal Clinical Guidelines Team (UK) (2014). National Institute for Health and Care Excellence: clinical guidelines. Gallstone disease: diagnosis and management of cholelithiasis, cholecystitis and choledocholithiasis. National Institute for Health and Care Excellence, London. https://www.nice.org.uk/guidance/cg188/evidence/full-guideline-pdf-193302253. Accessed Mar 2022 25473723

[2] Overby DW, Apelgren KN, Richardson W, Fanelli R (2010). SAGES guidelines for the clinical application of laparoscopic biliary tract surgery. Surg Endosc.

[3] AUGIS/ASGBI J (2015) Pathway for the management of acute gallstone diseases. https://www.augis.org/Portals/0/Guidelines/Acute-Gallstones-Pathway-Final-Sept-2015.pdf. Accessed Mar 2022

[4] Baucom R, Feurer I, Shelton J, Kummerow K, Holzman M, Poulose B (2016). Surgeons, ERCP, and laparoscopic common bile duct exploration: do we need a standard approach for common bile duct stones?. Surg Endosc.

[5] Sallam M, Nassar AHM, Kilpatrick R, Ali K (2021). A proposed difficulty grading system for laparoscopic bile duct exploration Benefits to practice and research. Br J Surg.

[6] Nassar AHM, Ng HJ, Katbeh T, Cannings E (2022). Conventional surgical management of bile duct stones. A service model and outcomes of 1318 laparoscopic explorations. Ann Surg.

[7] Lopez-Lopez V, Gil-Vazquez PJ, Fereras D, Nassar AHM, Bansal VK (2022). Multi-institutional expert update on the use of laparoscopic bile duct exploration in the management of choledocholithiasis: lesson learned from 3950 procedures. JHBPS.

[8] Nassar AHM, Gough V, Hwei Ng, Katbeh T, Khan K (2023). Utilisation of laparoscopic choledochoscopy during bile duct exploration and evaluation of the wiper blade manoeuvre for transcystic intrahepatic access. Ann Surg.

[9] Fang L, Wang J, Dai WC (2018). Laparoscopic transcystic common bile duct exploration: surgical indications and procedure strategies. Surg Endosc.

[10] Schutz SM, Abbott RM, Schutz SM (2000). Grading ERCPs by degree of difficulty: a new concept to produce more meaningful outcome data. Gastrointest Endosc.

[11] Kim J, Cho JY, Han HS (2021). Validation of a difficulty scoring system for laparoscopic liver resection in hepatolithiasis. Surg Endosc.

[12] Enochson L, Sharp N, Gimberg K, Sandblom G (2020). The location of bile duct stones may affect intra- and postoperative cholecystectomy outcome: a population-based registry study. Am J Surg.

[13] Kao C, Seagar R, Heathcock D (2021). Factors that predict the success of laparoscopic common bile duct exploration for choledocholithiasis: A 10-year study. Surg Laparosc Endosc Percutan Tech.

[14] Suwatthanarak T, Akaraviputh T, Phalanusitthepha C (2021). Outcomes of laparoscopic common bile duct exploration by chopstick technique in choledocholithiasis. JSLS.

[15] Noble H, Whitley E, Norton Sand Thompson M (2011). A study of preoperative factors associated with a poor outcome following laparoscopic bile duct exploration. Surg Endosc.

[16] Lv S, Fang Z, Wang A, Yang J, Zhang W (2017). Choledochoscopic holmium laser lithotripsy for difficult bile duct stones. J Laparoendosc Adv Surg Tech A.

[17] Ma Z, Zhou J, Yao L, Dai Y (2022). Safety and efficacy of laparoscopic common bile duct exploration for the patients with difficult biliary stones: 8 years of experiences at a single institution and literature review. Surg Endosc.

[18] Li M, Tao Y, Shen S (2020). Laparoscopic common bile duct exploration in patients with previous abdominal biliary tract operations. Surg Endosc.

[19] Griffiths EA, Hodson J, Vohra RS, Marriott P, Katbeh T, Zino S, Nassar AHM, CholeS Study Group (2019). West midlands research collaborative. Utilisation of an operative difficulty grading scale for laparoscopic cholecystectomy. Surg Endosc.

[20] Navaratne L, Martinez IA (2021). Transductal versus transcystic laparoscopic common bile duct exploration: an institutional review of over four hundred cases. Surg Endosc.

[21] Jones T, AlMusawi J, Navaratne L (2019). Holmium laser lithotripsy improves the rate of successful transcystic laparoscopic common bile duct exploration. Langenbecks Arch Surg.

