# Supplementary material for: A proposed difficulty grading system for laparoscopic bile duct exploration: benefits to clinical practice, training and research
Source: Surg Endosc. 2023 Jun 22;37(9):7012–23. doi: 10.1007/s00464-023-10169-9 (PMC10462500; doi:10.1007/s00464-023-10169-9)
Supplement: Supplementary file 1 — Supplementary file1 (DOCX 21 KB) [file 464_2023_10169_MOESM1_ESM.docx]

***Supplemental Table 1: Procedural difficulty, exploration approach, operative and postoperative outcome parameters in patients with and without a history of previous ERCP in 1335 LBDE.***

|  | Previous ERCP  No=108 (8%) | No previous ERCP  No=1227 (92%) | P value | OR (95% CI) |
| --- | --- | --- | --- | --- |
| Easy (Grade I A,B & II A,B) | 46 (42.6%) | 913 (74.4%) | **<0.001** | 0.255 (0.171, 0.382) |
| Difficult (Grade III, IV, V) | 62(57.4%) | 314 (25.6%) | **<0.001** | 0.255 (0.171, 0.382) |
|  |  |  |  |  |
| Exploration approach |  |  |  |  |
| Transcystic | 34 (31.5%) | 853 (69.5%) | **<0.001** | 0.201 (0.132, 0.308) |
| Choledochotomy | 74 (68.5%)) | 374 (30.5%) | **<0.001** | 4.964 (3.249, 7.584) |
| Choledochscopy utilisation | 97 (89.8%) | 857 (69.8%) | **<0.001** | 3.807 (2.017, 7.187) |
|  |  |  |  |  |
| Biliary drainage | 67 (62%) | 475 (38.7%) | **<0.001** | 2.753 (1.836, 4.130) |
|  |  |  |  |  |
| Median Operative time (mins) | 140 (IQR 115 – 184) | 100 (IQR 75 – 135) | **<0.001** | - |
|  |  |  |  |  |
| Conversion to open | 1 (0.9%) | 15 (1.2%) | 0.786 | 0.755 (0.099, 5.772) |
|  |  |  |  |  |
| Median Total Hospital stay* | 9 days (IQR 6 – 13) | 7 days (IQR 4 – 13) | **<0.001** | - |
|  |  |  |  |  |
| Resolved in one episode | 30 (27.7%)** | 895 (72.9%) | **<0.001** | 0.143 (0.092, 0.221) |
|  |  |  |  |  |
| Presentation to resolution <2 weeks | 22 (20.3%) | 765 (62.3%) | **<0.001** | 0.154 (0.095, 0.250) |
|  |  |  |  |  |
| * Including all hospital admissions, ** surgery during same admission as ERCP | | | | |
