# Supplementary figures and images for: A proposed difficulty grading system for laparoscopic bile duct exploration: benefits to clinical practice, training and research
Source: Surg Endosc. 2023 Jun 22;37(9):7012–23. doi: 10.1007/s00464-023-10169-9 (PMC10462500; doi:10.1007/s00464-023-10169-9)

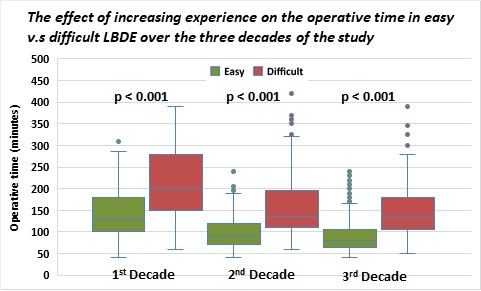

Supplement: Supplementary file 2 — Supplementary file2 (JPG 45 KB) Supplemental Fig. 1 The effect of increasing experience on the operative time in easy v.s difficult LBDE over the three decades of the study [file 464_2023_10169_MOESM2_ESM.jpg]
